# Intraprofessional collaboration and learning between specialists and general practitioners during postgraduate training: a qualitative study

**DOI:** 10.1186/s12913-016-1619-8

**Published:** 2016-08-11

**Authors:** Loes J. Meijer, Esther de Groot, Mirjam Blaauw-Westerlaken, Roger A. M. J. Damoiseaux

**Affiliations:** 1 University Medical Center Utrecht, Julius Center for Health Science and Primary Care, Broederplein 43, Zeist, 3703 CD The Netherlands; 2 University Utrecht Master Educational Sciences, Utrecht, The Netherlands

**Keywords:** Intraprofessional learning, Intraprofessional collaboration, Shared patient care, Postgraduate education, Interviews, General practitioner trainees, Specialist trainees

## Abstract

**Background:**

During postgraduate training, general practitioners and other specialists must learn how to deliver shared care to patients; however, the development of formal *intra*professional education is often hampered by curricular constraints. Delivering shared care in everyday work provides trainees with opportunities for informal learning from, about and with one another.

**Methods:**

Twelve semi-structured interviews were undertaken with trainee general practitioners and specialists (internal medicine or surgery). A thematic analysis of the input was undertaken and a qualitative description developed.

**Results:**

Trainees from different disciplines frequently interact, often by telephone, but generally they learn in a reactive manner. All trainees are highly motivated by the desire to provide good patient care. Specialist trainees learn about the importance of understanding the background of the patient from GPs, while GP trainees gain medical knowledge from the interaction. Trainees from different disciplines are not very motivated to build relationships with each other and have fewer opportunities to do so. Supervisors can play an important role in providing *intra*professional learning opportunities for trainees.

**Conclusions:**

During postgraduate training, opportunities for *intra*professional learning occur, but there is much room for improvement. For example, supervisors could increase the involvement of trainees in collaborative tasks and create more awareness of informal learning opportunities. This could assist trainees to learn collaborative skills that will enhance patient care.

**Electronic supplementary material:**

The online version of this article (doi:10.1186/s12913-016-1619-8) contains supplementary material, which is available to authorized users.

## Background

Collaboration between medical professionals from different disciplines working in separate organisations is essential to provide good-quality patient care [1]. Studies of intraprofessional collaboration (IPC) between qualified specialists and general practitioners have shown that collaboration is not always effective [2, 3]. Thus, it would appear that medical professionals could benefit from learning shared patient care skills during their training, which they can then transfer to their professional practice [4].

It is often difficult to incorporate formal intraprofessional education (IPE) into the curriculum [5–7]. In some institutions, integrated care rotation takes place, for example in Canada, where psychiatry residents undergo co-training in community care and primary care. However, implementation in most residency programmes remains difficult [7]. IPE often occurs in an implicit and unstructured manner [8]. Physicians do not seem to be convinced of the urgency to incorporate training in these skills into the curriculum [5, 9]. In addition, training in these general skills needs to be embedded within the formal structure of education in each specific discipline, but this may require complex adjustments to the curriculum [10, 11]. Therefore, it is of importance to explore whether and how general practitioner trainees (GP trainees) and specialist trainees work together to deliver shared care, what role qualified specialists and GPs play during their training and whether trainees value these moments of collaboration as learning opportunities. On this basis, informal intraprofessional learning (IPL) might be improved, leading to the enhancement of shared care delivery by specialists and general practitioners.

### Work-related intraprofessional learning

Medical professionals deliver shared care in various ways, differing in the intensity of interaction and the level of interdependence, both of which influence how and whether IPL occurs [12, 13]. The workplace learning model developed by Tynjälä distinguishes between learning processes (how something is learned), outcomes (what is learned) and the factors that affect learning [14]. In addition, learning processes may be formal or informal: Marsick and Watkins define formal learning as institutional and highly structured, and informal learning as less structured, with the student primarily responsible for the learning process [15]. Informal learning may be reactive, deliberate or implicit [4]. Reactive learning involves students noting facts and opinions, asking questions and observing the effects of actions. In deliberate learning, students discuss and review past actions and experiences, while in implicit learning, students are not aware of the learning process. The potential outcomes of learning processes are diverse [14, 16], and in a healthcare setting, in particular, workplace learning could contribute to better collaboration, more understanding of each other’s roles and responsibilities, greater clinical knowledge and/or improved levels of communication [6, 12, 17].

### Facilitators of and barriers to workplace learning

What and how students learn depends on facilitators and barriers related to the student and to the learning context, where the motivation of the student is an important facilitator [13, 18]. Studies have shown that the motives behind qualified GPs and specialists working in collaboration are concern for their patients, getting to know each other, the prevention of unnecessary referrals and the transfer of knowledge [2, 19]. Specialists prefer incidental, professional and personal interactions with GPs, while GPs mention building relationships, mutual respect, exchange of knowledge and being inspired as motives for collaboration [19, 20]. Management support has also been indicated to be essential for IPC [21].

Barriers to IPC may be related to work pressure. When the time and space to think and critically discuss issues with peers are lacking, deliberate learning does not take place [22, 23]. Another barrier to IPC may relate to difference in status, whether this is real or perceived: specialists are frequently considered to have a higher status than GPs [24, 25]. Specialists have also indicated that they do not consider GPs to be equals, and they also believe that they would learn little from them [2, 26].

### Research questions

Above we described studies of qualified GPs and specialists involved in collaboration in medical practice. What is not clear is whether trainees experience the same facilitators of and barriers to IPC. This is highly relevant, especially when considering options to use IPC for IPL support. In this study we evaluate whether and how IPC takes place for GP and specialist trainees and how trainees learn from professionals of another medical discipline. The research questions we aim to answer are:

1. What types of learning (formal, implicit, reactive or deliberate) occur during the delivery of shared care by trainees?
2. What do GPs and specialist trainees learn from delivering shared care?
3. What are the facilitators of and barriers to the intraprofessional learning (IPL) of trainees?

## Methods

### Study setting

In the Netherlands, postgraduate training does not provide formal education in skills that improve learning from and about each other while working together, with the aim of enhancing shared care delivery. During their second year, GP trainees take several internships within the hospital setting, for example, in the emergency department. However, for the remainder of their postgraduate studies, GP trainees and specialist trainees do not work in the same location: specialist trainees work in the hospital and GP trainees work in general practice.

### Participants

We used a convenience sample of twelve trainees from the same region in the Netherlands (see Table 1). All participants were recruited through the network of one of the researchers (LM). The participants were unknown to the researcher who conducted the interviews (MB).

**Table 1:** Characteristics of participants

	Professional role	Age	Gender	Year of training
1	GP‛ trainee	34–36	F*	3rd
2	GP trainee	31–33	F	3rd
3	GP trainee	28–30	M^	3rd
4	GP trainee	28–30	F	3rd
5	GP trainee	34–36	M	3rd
6	GP trainee	28–30	F	3rd
7	Specialist trainee: surgery	28–30	F	2nd
8	Specialist trainee: surgery	28–30	M	2nd
9	Specialist trainee: surgery	28–30	F	3rd
10	Specialist trainee: internal medicine	28–30	M	2nd
11	Specialist trainee: internal medicine	34–36	M	4th
12	Specialist trainee: internal medicine	34–36	F	6th

* F female *^ M* male *‛GP* general practitioner

### Data collection

Based on our theoretical framework, we developed a semi-structured individual interview format with open-ended questions divided into four themes (see Table 2 and Additional file 1). Pilot interviews were undertaken with trainees from a different region and with a GP staff member.

**Table 2:** Sample questions from the interviews^b^

Theme	Example question
Forms of intraprofessional collaboration	At what moments in time and in what ways did you interact with medical specialists^a^ during your training?
Formal and informal learning activities during intraprofessional collaboration	Do you encounter learning opportunities during your interactions with other specialists, and can you give examples? Does this influence your actions?
Learning outcomes of intraprofessional collaboration	What does collaboration with other specialists give you?
Barriers and facilitators	What hinders or prevents you from collaborating with a specialist (in training)?

^a^Specialist here also refers to General Practitioners

^b^Additional file 1: the Interview guide

### Design and procedure

A general qualitative methodology was chosen, using interviews to obtain detailed information about the perceptions of trainees. A week before the interview, participants received information about the study. At the beginning of each interview, the concepts of IPC and IPL were explained to ensure a common understanding. All interviews (30 min on average) were performed by one of the researchers (MB), and they were audiotaped and fully transcribed.

### Data analysis

The transcriptions of the interviews were analysed (using NVivo) in several rounds. In the first round, one researcher (MB) selected all of the relevant excerpts and developed a coding scheme. Using this first scheme, two interviews were independently coded by three researchers (MB, EdG, LM): two education specialists and one general practitioner. The three researchers then compared codes and adapted the coding scheme after discussion. They then coded three interviews using the adapted scheme. After comparisons, the final coding scheme was determined.

Subsequently, one researcher (MB) coded all of the remaining transcripts and, when in doubt, the code was discussed. After coding the first eight interviews, the scheme remained stable, indicating that saturation would be feasible with twelve respondents. In the third round, one researcher (MB) compared the results with those from studies of qualified specialists, reporting unexpected findings. All of the researchers discussed this analysis and determined whether learning should be considered implicit, reactive or deliberate. Situations in which other people were contacted for a learning purpose were seen as deliberate learning. When others were contacted primarily to discuss a patient, this was considered reactive learning. Finally, themes derived inductively from the data were discussed by the team and the excerpts from the transcripts were subsequently translated into English.

### Ethical considerations

Ethical approval for the project was granted by the Ethical Review Board of the Dutch Organisation for Medical Education. Written consent for extensive use of the audiotape recordings was obtained from the participants, who were informed that their participation was on a voluntary basis; that their responses would be anonymised; and that they were free to withdraw from the study at any time without explanation.

## Results

### Intraprofessional collaboration between trainees

Trainees interact with professionals from different medical disciplines working in other organisations on a daily basis. GP trainees frequently interact with specialists by telephone or letter to discuss patients and make referrals, to elucidate ambiguous decisions and to ask for advice. Specialist trainees, in turn, send letters to GPs after each of their patients is discharged from hospital, but they do not often call GPs. When they do call, their questions concern the background of a patient:

*I call when I have additional questions when there is a dilemma … a patient is in a palliative process within terminal care … then I also call. … I am unsure whether that would be called often or not, I think not very often*.

Specialist trainees regularly meet qualified GPs face to face during multidisciplinary meetings in the hospital, to which GP trainees are not invited. GP trainees deliver shared care with specialist trainees when they are interns in the emergency department. Specialist trainees do not rotate with general practice interns.

### Formal and informal learning

GP trainees meet qualified specialists as teachers during their postgraduate training. In the hospital, meetings only occur with qualified GPs. The trainees mentioned that formal IPE does not take place, but suggested it primarily occurs informally in the context of patient care. GP trainees learn from and about specialists (qualified or trainees) by asking questions and discussing issues on the telephone. If they are certain about their diagnosis, interactions with specialists are brief, with little learning occurring. They suggest that they may learn more from their discussions with a specialist when they are less certain:

*Yes, when I am very sure … they are very inclined to follow my judgement. But when I start the conversation with a question [such as] ‘I would like to discuss this with you’, that is another approach, then I do learn for sure*.

When in doubt, they call a specialist:

*… patients who are reluctant to change to tablets, when they have been told by a specialist that they should be given injections. And therefore I just called a specialist once [and asked] ‘How would you proceed with these patients because we have these patients coming from you and in our guidelines it is said that … What is your advice*?

They learn from listening to an expert on the telephone:

*You have a lack of knowledge sometimes and they teach you*.

They ask and receive feedback face to face, by telephone or through specialists’ letters:

*When you have referred a patient and you receive a letter explaining what was the matter, yes, well, of course you learn whether you were right or wrong*.

Specialists trainees learn from GPs through questioning. Surgery and internal medicine trainees consider GPs to be experts in understanding the background of the patient:

*When you are in a difficult situation, then it is just very nice to talk with the GP about it. I had a patient with a wish for euthanasia for example. Then it is a real comfort to talk about this with the GP who knows that patient incredibly well … whether they think the wish is sincere at that moment or that other factors are at play*.

Specialist trainees learn from discussions with qualified GPs at multidisciplinary meetings in the hospital or during phone calls with qualified GPs:

*Sometimes we are not sure whether a patient needs to come to the hospital immediately or whether a regular consultation is OK. With acute vascular disease this occurs very often … this is something you discuss, and together you come to the best solution*.

Specialist trainees do not mention asking for feedback but they consider GP referral letters a good source of information:

*Well, the GP’s referral with the prior history of that patient. And what the GP has done already, yes, that may very much guide you.*

### Outcomes of learning

All participants agreed that good patient care involves shared patient care. As one GP trainee summarised:

*Good medical care is about crossing borders. When the GP is not informed about the actions of the specialist, you as a GP are not able to provide good patient care. And at the same time, a specialist needs to know from the GP what other issues have to be considered. This is very important, especially for more complex problems*.

Learning is considered a spin-off of these exchanges.

GP trainees indicated that delivering care with other medical professionals brings them new medical knowledge. A lot can be learned when a specialist has the time and the inclination to explain the problem:

*Which situations may or may not become problematic. What does or does not relate to the disease and what is to be expected. … Things you rarely see as a GP, but a specialist [sees] more often.*

Through feedback or referral letters, GP trainees learn why specialists act the way they do. Collaboration gives GP trainees experience with good patient care processes and leads to better communication with specialists:

*For example, that you should start with a specific question and start your story afterwards. Instead of letting them hear the whole story, without them knowing where to focus, and then ask your question at the end. So I learned a bit about the way to do this – what is the most optimal way of communicating.*

What is striking is that GP trainees do not mention situations in which the learning outcome is ‘getting to know each other’, although they indicate that IPC is easier when you know the name and the face of the other person.

According to specialist trainees, interactions with GPs first and foremost lead to the learning outcome of ‘information about patients and their background’, which often affects their actions. Specialist trainees think that good collaboration and improved communication are valuable learning outcomes:

*In communication with a GP I have a lot to learn. The questions I asked, the answers I received – in the beginning they didn’t match. I have found my way now. Little by little I have learned from and with those GPs how I should do this*.

Some interviewees mentioned improved understanding of why GPs act the way they do. Finally, specialist trainees also indicated that knowing one another professionally makes collaboration easier:

*… some GPs are very versatile in small operations or have a background in surgery. Yes, that is nice to know because you have a completely different conversation then*.

### Facilitators of and barriers to IPL

For both groups of trainees, supervisors are important facilitators of learning. Most GP trainees indicated that their supervisor was a role model for them, and that their supervisor encouraged them to collaborate with specialists:

*Supervisors will say very readily ‘Consult a specialist then’. They do not want you to struggle on your own*.

Specialist trainees also mentioned that their supervisors stimulated them to contact GPs in a friendly and positive manner:

*In recent years I have noticed that my supervisors are very keen on good communication with GPs. They say: ‘Send a letter to the GP’ or ‘Mention this to the GP’, we get that kind of remark very often*.

Specialist trainees mentioned that collaboration with GP trainees depends on and is influenced by having a pager. When they have a pager, trainee specialists are frequently contacted by GPs about patients to be referred, or for discussions about patients. It is precisely those moments of interaction that are valuable learning opportunities, especially when they have a pager during the day, when most consultations take place. When they do not have a pager, or during the night, specialist trainees miss out on opportunities for learning from conversations with GPs. Surgery trainees have a pager during the day more often than do internal medicine trainees. However, the pager also increases work pressure:

*When you have a pager, you are called very frequently … you just do not have the time to really think about what someone is saying*.

The main barriers to IPL are work pressure and logistics. Some GP trainees feel that busy specialists are not inclined to explain things:

*Then they do not really have the opportunity to talk with you. It hinders you sometimes. You get the feeling they are not giving their full attention to the conversation*.

Opportunities for direct contact depend on accessibility, as it seems to be difficult for both groups to reach one another by phone. In this respect, location is an important factor, as being in close proximity or being perceived to be so makes a difference:

*A hospital is a kind of world of its own and all the people within the hospital communicate with each other very easily. Here in X, everyone knows one another from meetings, but GPs, well, they are a group from outside. You do see GPs in the emergency departments, but actually we are in different worlds. And we rarely see GPs face to face*.

Thus, being in different locations means face-to-face interaction is rare, making the learning of skills by modelling impossible. Nevertheless, both groups of trainees said that interrelationships between GPs and specialists are good and generally pleasant.

## Discussion

General practitioners and specialists deliver shared patient care: they have clear roles and tasks, and they interact in relation to referrals, when they have various questions or in discussions related to patient care.

### Learning during intraprofessional collaboration

Our results show that trainees learn informally during activities necessary for patient care. Trainees from all of the disciplines studied seek interaction with each other and with qualified professionals to resolve problems or obtain feedback. Very often they lack time for reflection and they have to make decisions quickly, which indicates reactive learning [16]. Elsewhere, it has also been found that medical professionals do not very often learn deliberately while at work [27]. For deliberate learning to occur, reflection is important [15], which probably occurs less often because of work pressure, as is true for both qualified professionals and trainees. Trainees have something to gain because they are explicitly expected to learn during their training. If they reflected more on their existing interactions, more could be learned.

### Learning outcomes

IPC leads to a better understanding of one another’s professional roles, responsibilities and behaviour, for GP trainees as well as for specialist trainees. In addition, it leads to better collaboration and better communication. This is in concordance with the literature on qualified physicians [12, 17]. GP trainees gain knowledge about clinical topics, while specialist trainees mainly learn about patients and their background. IPC may contribute to the learning outcome of’getting to know each other’, but this was not mentioned by trainees in this study. It is possible that getting to know each other is easier during face-to-face contact [28] and that is precisely what trainees have little opportunity for. Thus, they miss out on acquiring relational skills that they will need later in their careers.

### Facilitators of and barriers to learning

Contributing to high-quality patient care and building relationships are important motives for the IPL of physicians working in practice [19, 28]. Indeed, contributing to patient care was seen as the main motivating factor for trainees. In contrast, the need to build relationships with others was not mentioned. Several trainees indicated that building relationships with one another would only become important once they were qualified physicians themselves and had worked for a longer period in the same hospital.

Organisational structures were indeed an important facilitating factor in terms of whether trainees have the opportunity to interact and are encouraged to interact with other professionals. For example, if GP trainees joined their supervisors in multidisciplinary meetings at the hospital this would help them build a network with both specialists and specialist trainees. Trainees could thereby learn from, with and about each other’s profession. A pager provides another opportunity for learning, but having time to reflect during actual calls is important if they are to become learning tools.

Supervisors could provide more support to their trainees in IPC and in learning from such collaboration. For example, supervisors of specialist trainees could encourage them to engage in conversation with GP trainees during the latter’s internship in the emergency department and to learn about their work as a GP and how they might collaborate in the future.

External pressure creates a barrier to IPL for trainees. In contrast, hierarchy does not seem to be a barrier. It has been suggested that in recent years the medical hierarchy has diminished [29]. Several respondents spoke about improvements, using expressions such as ‘these days’ or ‘in recent years’. Finally, GP trainees learn a lot about the daily practice of specialists during their emergency care internship. For specialist trainees to learn about the work of GPs, an internship in a GP practice would obviously be of benefit.

### Limitations and future research

Our study took place in a non-academic hospital with complete ‘in-house’ diagnostics, a high volume of care and a central position in the regional healthcare system. It is possible that our results cannot be generalised to trainees in academic hospitals, where specialists do not know one another very well, or to trainees working in small local hospitals, where GPs perhaps belong to a smaller circle of professionals taking care of patients.

In our study we have seen that trainees have many opportunities for IPC which could be used for learning. How these opportunities can best be exploited requires further study. For example, a study determining how supervisors can best help trainees with reflection on intraprofessional collaboration would be valuable.

Trainees differ from qualified physicians with respect to opportunities for direct contact with other professionals. Specialist trainees are not always given a pager, but as a pager appears to enable learning, it would be of value to undertake observational studies examining how they might be better used as learning tools.

## Conclusions

Our study explored types of intraprofessional learning that occur during the delivery of shared care between postgraduate trainees in general practice, internal medicine and surgery. Their experiences are in most cases comparable with the experiences of qualified physicians already described in the literature. The competences necessary for intraprofessional collaboration could be learned more formally by using co-training, with specialist trainees spending time in general practice and GP trainees in a hospital setting [30]. However, these competences may in part be learned informally and collaboratively during the delivery of shared care. Making trainees and supervisors more aware of the opportunities for intraprofessional learning during shared care, with other professionals and trainees from other disciplines, could assist them to learn in a more deliberate manner which will ultimately benefit patient care.

## Abbreviations

GP trainees, General Practitioner trainees; IPC, intraprofessional collaboration; IPE, intraprofessional education; IPL, intraprofessional learning

## Additional file

Additional file 1: Interview guide: Interviews with trainee general practitioners and specialists. (DOCX 15 kb)
