# Supplementary material for: Intraprofessional collaboration and learning between specialists and general practitioners during postgraduate training: a qualitative study
Source: BMC Health Serv Res. 2016 Aug 11;16:376. doi: 10.1186/s12913-016-1619-8 (PMC4982222; doi:10.1186/s12913-016-1619-8)
Supplement: Additional file 1: — Interview guide: Interviews with trainee general practitioners and specialists. (DOCX 15 kb) [file 12913_2016_1619_MOESM1_ESM.docx]

**Additional file 1: Interview guide: Interviews with trainee general practitioners and specialists**

*Theme 1: Forms of intraprofessional collaboration during training*

1. At what moments in time and in what ways did you interact with hospital specialists and general practitioners during your training?
   1. Do you miss anything regarding this?

E.g. During referrals: letter, telephone, e-mail, face-to-face

1. At what moments do you formally work with a specialist/general practitioner during your postgraduate training?

*Theme 2: Formal and informal learning activities during intraprofessional collaboration*

1. Do you feel that interaction and collaboration with specialists/general practitioners is needed to be able to offer good care to patients?
   1. At what moments do you consciously seek to make contact with a specialist/general practitioner, and how do you go about this?
2. Do you experience learning moments in the interaction with specialists/general practitioners, and can you describe these?
   1. What other opportunities for being able to learn something from specialists/general practitioners do you experience? How consciously are you engaged in this?
   2. Has your contact with the other line changed the way you act, or have you gained something from it that you can use at other moments?

*Theme 3: Learning outcomes of intraprofessional collaboration*

1. What does interaction or collaboration with specialists/general practitioners give you?

E.g. mutual understanding of each other’s role and responsibilities, better cooperation, increased knowledge of clinical subjects/skills, better mutual relationships.

- 1. Do you feel that you and the specialists are aiming for the same goal? Or does it feel more like you are each doing your own thing?

*Theme 4: Factors that stimulate you or hold you back*

1. What stimulates you to collaborate with specialists/general practitioners?

E.g. caring for patients, personal relationships, mutual inspiration, knowledge transfer.

- 1. What would you really like to learn from specialists/general practitioners?
  2. What do you think that specialists and general practitioners could learn from each other?

1. Do you feel that intraprofessional collaboration is encouraged during your training?
   1. What role do your teachers/trainers play in this?
2. What hinders you/holds you back in the collaboration with specialists/general practitioners?
   1. What are the mutual relationships like?

Is there a hierarchy or equality?
